# Construction of Hierarchical-Targeting pH-Sensitive Liposomes to Reverse Chemotherapeutic Resistance of Cancer Stem-like Cells

**DOI:** 10.3390/pharmaceutics13081205

**Published:** 2021-08-05

**Authors:** Shuang Ba, Mingxi Qiao, Li Jia, Jiulong Zhang, Xiuli Zhao, Haiyang Hu, Dawei Chen

**Affiliations:** 1 School of Pharmacy, Shenyang Pharmaceutical University, No. 103, Wenhua Road, Shenyang 110016, China; bajunjie@163.com (S.B.); qiaomingxi@163.com (M.Q.); zjl1160@163.com (J.Z.); raura3687yd@163.com (X.Z.); haiyang-hu@hotmail.com (H.H.); 2 Department of Pharmacy, Heze Medical College, Heze 274000, China; jiali0978@163.com

**Keywords:** TPP conjugate, hierarchical targetability, cancer stem-like cells

## Abstract

Cancer stem-like cells (CSLCs) have been considered to be one of the main problems in tumor treatment owing to high tumorigenicity and chemotherapy resistance. In this study, we synthesized a novel mitochondria-target derivate, triphentlphosphonium-resveratrol (TPP-Res), and simultaneously encapsulated it with doxorubicin (Dox) in pH-sensitive liposomes (PSL (Dox/TPP-Res)), to reverse chemotherapeutic resistance of CSLCs. PSL (Dox/TPP-Res) was approximately 165 nm in size with high encapsulation efficiency for both Dox and TPP-Res. Cytotoxicity assay showed that the optimal synergistic effect was the drug ratio of 1:1 for TPP-Res and Dox. Cellular uptake and intracellular trafficking assay indicated that PSL (Dox/TPP-Res) could release drugs in acidic endosomes, followed by mitochondrial targeting of TPP-Res and nucleus transports for Dox. The mechanisms for reversing the resistance in CSLCs were mainly attributed to a synergistic effect for reduction of mitochondrial membrane potential, activation of caspase cascade reaction, reduction of ATP level and suppression of the Wnt/β-catenin pathway. Further, in vivo assay results demonstrated that the constructed liposomes could efficiently accumulate in the tumor region and possess excellent antineoplastic activity in an orthotopic xenograft tumor model with no evident systemic toxicity. The above experimental results determined that PSL (Dox/TPP-Res) provides a new method for the treatment of heterogenecity tumors.

## 1. Introduction

Tumors are composed of a heterogeneous population of cells [1,2,3]. Only a small subpopulation of cells, cancer stem-like cells (CSLCs), has self-renewal and tumor formation potential, while the majority of tumors consist of non-tumorigenic cells. CSLCs show a self-renewal capacity, differentiation potential invasiveness and resistance to traditional chemotherapy and radiotherapy, which contribute to tumor progression and therapy resistance [4]. Drug resistance of CSLCs has attracted increasing attention with the development of advanced treatment methods in recent years [5,6,7,8].

However, drug resistance of CSLCs are complex, and is associated with the overexpression of adenosine triphosphate (ATP)-binding transmembrane efflux pumps, enhanced DNA repair ability, inhibited apoptosis, self-renewal capacity and multilineage differentiation potential [9,10,11]. Multiple drug resistance (MDR) mechanisms described above are interrelated, leading to CSLCs being able to escape conventional chemotherapy methods. To solve this problem, chemotherapy drugs combined with MDR modulators have been investigated to achieve synergy and concurrently combat the CSLC-rooted heterogeneity [12]. More importantly, in consideration of their distinct physicochemical properties, pharmacokinetics profiles and anticancer mechanisms, it may be important to localize the delivery of the two or more agents in a single nanoparticle to achieve an optimal combination efficacy [13,14,15].

Another solution is to increase targeting accuracy of various ligands and targeting strategies [16]. Mitochondria are important subcellular organelles that play a crucial role in many biological processes. Mitochondria in cancer cells are structurally and functionally different from their normal counterparts. They exhibit extensive metabolic abnormalities and are more susceptible to interference than normal cell mitochondria [17]. Moreover, MDR of cancer cells contributes to insensitivity of mitochondria to apoptosis signals. Consequently, induction of apoptosis through directly targeting the mitochondria of cancer cells may be a strategy to avert drug resistance [18,19]. Sufficient lipophilicity and delocalization of the positive charge are the prerequisites for interaction of ligands with mitochondrial membrane which is known to have a large transmembrane potential. Taking advantage of this feature, many ligands such as protein transduction domains (PTDs), mitochondrial targeting sequences (MTSs), and lipophilic cations loading or conjugating with small molecules or nanoparticle drug delivery systems have been reported [20,21,22,23]. However, surface positive charges after cation modification result in off-target and nonspecific toxicity in vivo effects [24,25]. Therefore, it is important to enable the nanoparticles to aggregate in mitochondria after entering the cancer cells by utilizing the tumor cell microenvironment.

Resveratrol (Res), a natural polyphenolic compound, has a wide range of biological activities to inhibit the initiation, progression and metastasis of tumors [26]. Previous studies indicate that Res reverses resistance for cancer cells and CSLCs in multiple ways, such as preventing mitochondrial ATP synthesis, inhibiting Wnt/β-catenin signaling pathway and interfering with anti-apoptotic factors [27,28,29,30]. Targeted delivery Res to mitochondria where their redox properties and pro-apoptosis might be exploited to the maximum, can effectively improve the therapeutic efficacy of CSLCs [24,31,32].

**Figure 1:**
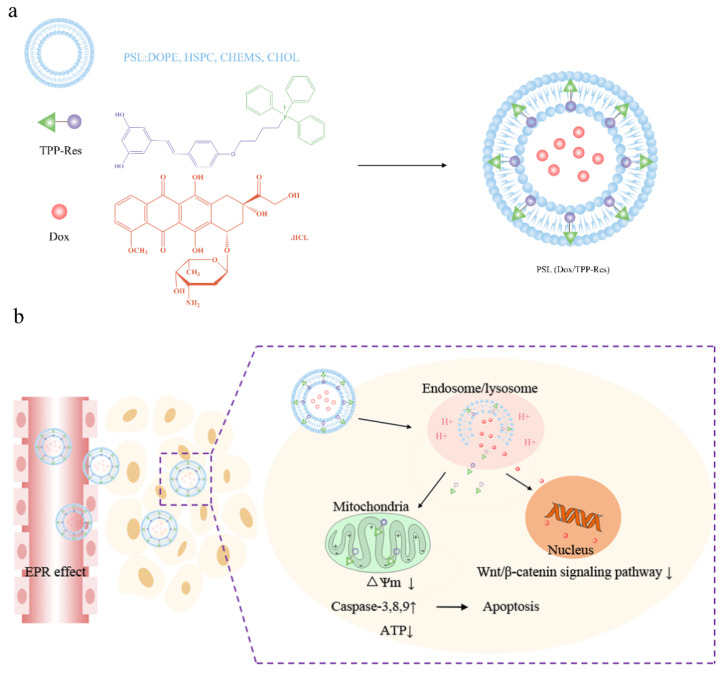
(**a**) Schematic of PSL (Dox/TPP-Res) composed of pH-sensitive liposome encapsulated with Dox and TPP-Res. (**b**) Schematic illustration of the hierarchical targeting strategy by PSL (Dox/TPP-Res) to reverse chemotherapeutic resistance in cancer stem-like cells.

Here we proposed a convergent therapeutic strategy using a hierarchical targeting delivery system to overcome obstacles to CSLCs-derived heterogeneity (Figure 1a). A novel mitochondria-target derivate, triphentlphosphonium-resveratrol (TPP-Res), was synthesized. TPP-Res and Dox were selected as the MDR modulator and cytotoxic drugs, which were co-loaded into different sites of the liposome with an optimized synergistic drug ratio. The liposomes were expected to fulfill several hierarchical tasks in cancer stem-like cells as shown in Figure 1b: (1) The liposomes accumulated in the tumor site under enhanced permeability and retention (EPR) effect-mediated passive targetability. (2) After initialization, the liposomes, which imparted a pH-sensitive function, was merged with the endosome membrane in the acidic environment of the endosome to expose the TPP-Res and Dox. (3) TPP-Res targeted mitochondria, triggered mitochondrial-mediated apoptosis and increased the accumulation of Dox in the nucleus to increase the therapeutic effects in CSLCs. The objectives of the present study were to prepare and characterize the co-loaded Dox and TPP-Res pH-sensitive liposomes. Cell cytotoxicity, cellular uptake, intracellular trafficking and the potential apoptosis-inducing effects on CSLCs were investigated. Finally, antitumor activity and biodistribution of the liposomes were assessed using an orthotopic xenograft CSLCs model.

## 2. Materials and Methods

### 2.1. Materials

Doxorubicin hydrochloride (Dox·Hcl) was purchased from Beijing HuaFeng United Technology Co., Ltd. (Beijing, China). Resveratrol (Res) was bought from Guan Jie Biotech (Xian, China). 1-Bromo-4-chlorobutane and triphenylphosphine (TPP) were obtained from Aladdin Co., (Shanghai, China). K_2_CO_3_ and NaI were supplied by Sinopharm Chemical Reagent Co., (Shanghai, China). Dioleoylphosphatidylethanolamine (DOPE) and hydrogenated soybean phospholipids (HSPC) were purchased from Shanghai A.V.T Technology Co., Ltd. (Shanghai, China). Cholesterol and cholesteryl hemisuccinate (CHEMS) were bought from J&K Scientific Co., Ltd. (Beijing, China). 3-(4,5-Dimethyl-thiazol-2-yl)-2,5-diphenyl-tetrazolium bromide (MTT), cell mitochondria isolation kit, mitochondrial membrane potential assay kit with JC-1, Caspase 3,8,9 assay kit, ATP assay kit, Lysotracker Green DND and Hoechst 33258 were purchased from Beyotime Biotechnology Co., Ltd. (Nantong, China). FITC anti-human CD44, FITC Mouse IgG1, PE anti-human CD24 and PE Mouse IgG2a were bought from BD Biosciences (Franklin Lakes, NJ, USA). β-Actin, β-catenin and cyclin D1 primary antibodies and corresponding horseradish peroxidase (HRP)-conjugated secondary antibodies were provided by Abcam (Cambridge, UK).

### 2.2. Cell Culture

MCF-7 human breast cancer cells (obtained from Chinese Academy of Medical Sciences, Beijing, China) were grown in DMEM medium (Gibco, Carlsbad, CA, USA) supplemented with 10% fetal bovine serum (FBS) and 100 U/mL penicillin and 100 μg/mL streptomycin (all from Sigma, St Louis, MO, USA) at 37 °C in 5% CO_2_ incubator.

For the culture of cancer stem-like cells (CSLCs), MCF-7 cells (2000 cells/mL) were cultured in 6-well ultralow attachment plate (corning, USA) and suspended in serum-free DMED-F12 medium (Gibco, Carlsbad, CA, USA) supplemented with 1 × B27, 20 ng/mL epidermal growth factor (EGF), 20 ng/mL basic fibroblast growth factor (bFGF), 0.4% (*w*/*v*) bovine serum albumin, 100 U/mL penicillin and 100 μg/mL streptomycin (all from Sigma, St Louis, MO, USA). After 10 days, CSLCs were harvested and grew as nonadherent spherical clusters of cells, named mammospheres. For further experimental use, mammospheres were collected by gentle centrifugation (800× *g*, 5 min), dissociated into single cells and then cultured to generate mammospheres of the next generation.

### 2.3. Animals

Female BALB/c nude mice (20 ± 2) g were supplied by Department of Experimental Animals, Shenyang Pharmaceutical University (Shenyang, China). Mice were acclimated at 25 °C and 55% of humidity under natural light/dark conditions with access to food and water ad libitum. All animal experiments were carried out in accordance with protocols evaluated and approved by the ethics committee of Shenyang Pharmaceutical University (the project identification code: SYPU-IACUC-C2018-12-7-102, date of approval: 7 December 2018).

### 2.4. Synthesis and Characterization of 4′-(4-O-Triphenylphosphoniumbutyl) Resveratrol (TPP-Res)

#### 2.4.1. Synthesis of 4-(4-O-Chlorobutyl) Resveratrol 

Resveratrol in DMF was subjected to reaction with K_2_CO_3_ (1.5 equiv) and 1-bromo-4-chlorobutane (1.5 equiv) for 24 h at room temperature. The mixture was extracted three times with ethyl acetate and 1 mol/L HCL. Abandoned water layer and the remaining organic layer were dried with MgSO_4_ and filtered. Thereafter, the solvent was removed under vacuum and the crude product was purified on silica gel chromatography using Ch_2_Cl_2_/ethyl acetate (85:15).

#### 2.4.2. Synthesis of 4-(4-O-Iodobutyl) Resveratrol

4-(4-O-chlorobutyl) resveratrol was dissolved in a saturated solution of NaI in acetone under reflux conditions. After 24 h, the mixture was extracted three times with ethyl acetate and water. Abandoned water layer and the remaining organic layer were dried with MgSO_4_ and filtered. After evaporation, the crude product was purified on silica gel chromatography using Ch_2_Cl_2_/ethyl acetate (9:1).

#### 2.4.3. Synthesis of TPP-Res

4-(4-O-iodobutyl) resveratrol and triphenylphosphine (5 equiv) were added in toluene under reflux conditions. After 6 h, the solvent was removed under vacuum and precipitated in excessive cold diethyl ether. The purified product was vacuum dried for three days to get rid of residual solvent.

#### 2.4.4. Characterization of Synthesized Products

The chemical structure of the intermediate products and TPP-Res was characterized by ^1^H-NMR spectra and ^13^C-NMR spectra (Bruker DRX-600) using DMSO-*d*_6_.

### 2.5. Preparation of the Co-Delivery Liposomes

Dox was encapsulated into the liposomes by ammonium sulfate gradient method and TPP-Res was incorporated by thin-film hydration method. Briefly, DOPE, HSPC, CHEMS, CHOL (2:1:1:1, *w*/*w*) were used as the lipid materials. Lipids and TPP-Res (10:1, *w*/*w*) were dissolved in ethanol. The organic solvents were removed by vacuum evaporator. The dried lipid films were hydrated in a 250 mM ammonium sulfate solution at pH 8.5 and sonicated with a probe-type sonicator at 200 w power (carried out every 1 s for a 3 s duration in ice bath) for 3 min. Then, the liposomes were consecutively extruded through a series of polycarbonate membrane filters with pore size of 800–200 nm. The external buffer was exchanged by eluting through a Sephadex G-50 column equilibrated with 10% sucrose, 25 mM Trizma base at pH = 8.5. Dox·Hcl (10:1, *w*/*w*) was added to the liposome and the mixture was incubated for 1.5 h at 37 °C. The unencapsulated drugs wereremoved by a Sephadex G-50 column with PBS (pH 7.4).

Dox liposome and TPP-Res liposome were prepared with the same method as mentioned above. Dir liposome (lipids: Dir = 100:1, *w*/*w*) were prepared as the same procedures as those of TPP-Res liposome.

### 2.6. Characterization of the Liposomes

Gel microcolumn method is a classic method to determine the encapsulation efficiency of liposomes. Liposomes were passed over a Sephadex G-50 column to remove the unencapsulated drugs. The amount of Dox was measured by a multifunctional microplate reader (Tecan, Austria) with excitation wavelength at 470 nm and emission wavelength at 585 nm. The amount of TPP-Res was determined by high performance liquid chromatography (HPLC) with detection wavelength at 306 nm. The mobile phase consisted of methanol and H_2_O (40:60, *v*/*v*). The encapsulation efficiency of liposomes was calculated using Equation (1).

(1) $$\mathrm{EE}\%=\frac{W_{encap}}{W_{total}}\times 100\%$$

where W*_encap_* was the measured amount of encapsulated drug, W*_total_* was the measured amount of feeding drug.

The particle size, size distribution and zeta potential of liposomes were analyzed by Zetasizer Nano ZS instrument (Malvern, UK) at 25 °C. Morphology of liposomes was observed using JEM-100CX transmission electron microscope (Jeol, Tokyo, Japan) with an accelerating voltage of 100 kV. Before visualization, a drop of each sample was first deposited on a carbon-coated copper grid. After 5 min, the grid was blotted with a filter paper to remove excess amount of solution. The copper grid was air dried and negatively stained with 2% phosphotungstic acid for 30 s.

In vitro release was performed by the dialysis method under a series of phosphate buffer saline (PBS, 0.01 M, pH 7.4, 6.5, 5.0). A volume of 2.0 mL liposomes in dialysis tubing (MWCO 8-14KDa) was immersed in 50 mL of the release medium and shook at a rate of 100 rpm at 37 °C. At different time points, 2.0 mL of release medium was taken and added equal volume of release medium. The concentration of Dox and TPP-Res in the release medium was determined by a multifunctional microplate reader and HPLC, respectively, as mentioned above.

### 2.7. Characterization of Cancer Stem-Like Cells (CSLCs)

To characterize the expression of cell surface markers, mammospheres were seeded at a density of 1 × 10^5^ cells per well in DMEM-F12 medium. MCF-7 or dissociated mammospheres were washed with cold PBS and stained with 5 µL anti-CD44-FITC and 5 µL anti-CD24-PE or with 5 µL isotype controls on ice for 30 min. After staining, the cells were washed with cold PBS three times and resuspended in 500 µL PBS. The samples were analyzed on a FACScan flow cytometer (BD Biosciences, Franklin Lakes, NJ, USA).

For limited dilution assays in vivo, a series concentration of MCF-7 or mammospheres (10^3^, 10^4^, 10^5^ cells/mL) were injected subcutaneously into axillary fossa of female BALB/c nude mice (n = 5). After 28 days, the presence of each tumor mass was observed after the inoculation [33].

### 2.8. In Vitro Cytotoxicity Assay

For cell cytotoxicity studies, MCF-7 (7 × 10^3^ cells/mL) and CSLCs (5 × 10^3^ cells/mL) were seeded in 96-well plates. After culturing for 24 h, cells were incubated with various drug formulations in their respective culture medium. After 48 h, 20 μL of MTT (5 mg/mL) was added into each well and further incubated for 4 h. Then we removed the medium and 150 μL DMSO was added to dissolve the formazan crystals. The absorbance was read on a multifunctional microplate reader at 570 nm. The cell survival percentages were calculated using Equation (2). The IC_50_ value of each sample was analyzed by SPSS 17.0 (Chicago, IL, USA). Equation (3) was used to calculate the resistance factor (RF) of CSLCs.

(2) $$\mathrm{Cell}\;\mathrm{viability}\;\left(\%\right)=\frac{A_{\mathrm{sample}}-A_{\mathrm{blank}}}{A_{\mathrm{control}}-A_{\mathrm{blank}}}\times 100\%$$

where A_control_ was the absorbance of cells in control, A_sample_ was the absorbance of cells in the presence of sample treatment and A_blank_ was the absorbance of medium.

(3) $$\mathrm{RF}=\frac{{\mathrm{IC}}_{50}\;\left(\mathrm{Dox}\right)}{{\mathrm{IC}}_{50}\;\left(\mathrm{Sample}\right)}$$

where IC_50_ (Dox) was the IC_50_ value of free Dox in CSLCs, IC_50_ (Sample) was the IC_50_ value of sample treatment in CSLCs.

### 2.9. Cellular Uptake of DOX

Fluorescence microscopy was used to observe the cellular uptake of various DOX-loaded formulations in CD44^+^ CSLCs. In brief, CSLCs were seeded at a density of 1 × 10^4^ cells/mL on 6-well culture plates in complete DMEM-F12 medium. After 24 h, different formulations with equivalent doses of Dox (10 μg/mL) and TPP-Res (10 μg/mL) were added to each well and further incubated for 6 h. The cells were washed three times with ice-cold PBS and stained with 5 µL anti-CD44-FITC on ice for 30 min. After staining, the cells were washed three times with ice-cold PBS 7.4 and fixed with 4% paraformaldehyde for 15 min. The fluorescent signal was imaged by a fluorescence microscopy (Olympus, Tokyo, Japan).

For flow cytometry analysis, CSLCs were seeded at a density of 1 × 10^5^ cells/mL on 6-well culture plates for 24 h. Different formulations were added into each well at a concentration of 10 μg/mL Dox and 10 μg/mL TPP-Res. After 6 h, the culture media were removed and the cells were washed three times with ice-cold PBS. The cells were then harvested by trypsinization, centrifuged at 1000 rpm for 5 min, resuspended in 500 μL of PBS medium and analyzed using FACScan flow cytometer (BD Biosciences, Franklin Lakes, NJ, USA).

### 2.10. Intracellular Transport

#### 2.10.1. Endosomal Escape

To investigate the subcellular transport of liposomes using confocal laser scanning microscopy (CLSM), CSLCs (1 × 10^4^ cells/mL) were cultured on microscope slides in a 6-well plate and incubated for 24 h. The cells were treated with different formulations for 0.5 h, 2 h and 4 h. The cells were then washed three times with ice-cold PBS and stained with LysoTracker green (60 min) to visualize endo-lysosomes. After being fixed with 4% paraformaldehyde for 30 min, the cells were stained with Hoechst 33258 for 10 min. The microscope images were captured using a FV1000-IX81 confocal laser scanning microscope (Olympus, Tokyo, Japan).

#### 2.10.2. TPP-Res Uptake in Mitochondrial Fraction

CSLCs were seeded at a density of 1 × 10^5^ cells/mL on 6-well culture plates for 24 h. Different formulations were added into each well at a concentration of 10 μg/mL Dox and 10 μg/mL TPP-Res. After 6 h, the culture media were removed and the cells were washed three times with ice-cold PBS. The cells were then harvested by trypsinization and we isolated the mitochondria by a cell mitochondria isolation kit. The mitochondrial fraction was resuspended in 500 µL of PBS medium and analyzed using FACScan flow cytometer (BD Biosciences, Franklin Lakes, NJ, USA).

### 2.11. Mechanisms of Apoptosis in CSLCs

#### 2.11.1. Mitochondrial Membrane Potential (ΔΨm) Detection

Mitochondrial membrane potential assay kit with JC-1(Beyotime, Nantong, China) was used to assess the change in mitochondrial membrane potential. CSLCs were seeded at a density of 1 × 10^5^ cells/mL on 6-well culture plates for 24 h. Then, negative control group (PBS), positive control group (CCCP) and different formulations at a concentration of 10 μg/mL Dox and 10 μg/mL TPP-Res were added into each well. After 4 h, cells were washed with PBS and stained with 500 mL of JC-1 (10 mg/mL) work solution for 20 min in dark place. After washing with PBS three times, the samples were analyzed with a multifunctional microplate reader (Tecan, Salzburg, Austria) at λ_ex_ (488 nm)/λ_em_ (590 nm) for red fluorescence or λ_ex_ (488 nm)/λ_em_ (530 nm) for green fluorescence. The obtained values were then expressed as average JC-1 red/green (R/G) signal intensity ratios.

#### 2.11.2. Caspase Activity Assays

The caspase activity test kit was used to measure the activities of caspase-3, 8 and 9. CSLCs were seeded at a density of 1 × 10^5^ cells/mL on 6-well culture plates for 24 h. Negative control group (PBS) and various formulations at a concentration of 10 μg/mL Dox and 10 μg/mL TPP-Res were added into each well. After 12 h, the cells were washed with PBS and lysed with lysis buffer for 15 min in an ice bath. The cell lysates were centrifuged at 10,000 rpm for 1 min at 4 °C. The supernatants were stored and treated with caspase-3, 8, 9 substrates, respectively. Caspase-3, 8, 9 activities were measured based on the absorbance values at 405 nm by a multifunctional microplate reader. The concentrations of total protein were measured by the Bradford method. pNA concentrations (nmol/g) were calculated according to the manufacturer’s instructions.

#### 2.11.3. ATP Contents Assay

To investigate the level of ATP in CSLCs, a luciferin/luciferase assay was carried out. A total of 1 × 10^5^ cells/mL of CSLCs were seeded into 6-well plates and incubated for 24 h. The cells were exposed to negative control group (PBS) and various formulations at a concentration of 10 μg/mL Dox and 10 μg/mL TPP-Res. After 12 h, the cells were washed three times with ice-cold PBS, solubilized in cell lysates and centrifuged (12,000× *g*) at 4 °C for 10 min. After centrifugation, the supernatant was collected and mixed with ATP monitoring solution. The luminescence value was measured by a multifunctional microplate reader. ATP contents were calculated according to the manufacturer’s instruction. Raw data were converted to ATP concentrations according to the standard calibration curve. The concentrations of total protein were measured by the BCA method.

### 2.12. Western Blotting

To study the inhibition of the Wnt/β-catenin signaling pathway, CSLCs (1 × 10^5^ cells/mL) were seeded in 6-well plates and cultured for 24 h. The liposomes and negative control group were subsequently added to each well. After 48 h, the cells were washed twice with ice-cold PBS and lysed by RIPA on ice for 30 min. The cell lysates were centrifuged at 16,000 rpm for 5 min and the supernatants were collected for determining with BCA protein assay kit. Each sample was adjusted to get an identical protein concentration. The samples of protein were resolved by 15% SDS-PAGE and then electroblotted onto polyvinylidene fluoride (PVDF) membranes. Blots were probed with primary antibodies (β-catenin, 1:5000; cyclin D1, 1:25; β-actin, 1:1000) overnight at 4 °C, followed by the horseradish peroxidase-conjugated secondary antibodies (HRP, 1:2000). Then, the proteins were visualized, exposed and photographed with MicroChemi 4.2 Gel Imager (DNR, Jerusalem, Israel).

### 2.13. In Vivo Biodistribution

To establish the xenograft tumor model, CSLCs (1 × 10^4^ cells/mL) suspended in 200 μg/mL PBS were inoculated subcutaneously into each female BALB/c nude mice. When the tumor reached approximately 50 mm^3^, free Dir and Dir-loaded liposome (100 μg/mL) were intravenously injected through a tail vein, respectively. After anesthesia with isoflurane, mice were imaged at 0.5 h, 1 h, 2 h, 4 h, 8 h, 12 h, 24 h, 36 h and 48 h with a Carestream FX PRO Image System (Carestream Health, Rochester, NY, USA). Eventually, the mice were sacrificed, and the major organs and tumors were harvested and imaged with the machine. The fluorescence intensities of tissues were analyzed using NIH Image J software.

### 2.14. In Vivo Antitumor Activities

Tumor-bearing mice were obtained from female BALB/c nude mice as described above. The mice were randomly divided into six groups (six mice per group) when the tumor reached 50 mm^3^. Mice were intravenously injected with various formulations every three days, three times. Dox, Res and TPP-Res administration dosage was 4 mg/kg. The mice were monitored every day for tumor progression and body weight. After 22 days, all mice were sacrificed and tumors were harvested and weighed.

### 2.15. Statistical Analysis

Quantitative data are presented as the mean ±standard deviation (SD) deviation from at least three independent experiments. Statistical comparisons were determined by the one-way analysis of variance (ANOVA) between ≥3 groups or Student’s *t*-test between 2 groups. A difference with *p* < 0.05 was considered to be statistically significant.

## 3. Results and Discussions

### 3.1. Characterizations of TPP-Res

TPP-Res was synthesized in three processes (Figure S1). Briefly, the oxhydryl of resveratrol was replaced by a chlorobutyl group which was then transformed to the TPP derivative via two consecutive nucleophilic substitution reactions: chlorobutyl-iodobutyl-triphenylphosphoniumbutyl. It was not advisable to carry out direct substitution of chloride by TPP, since it required a high temperature which led to some decomposition [34]. The chemical structure of TPP-Res and the intermediate products were confirmed by ^1^H-NMR spectra and ^1^^3^C-NMR spectra in Figures S2 and S3. The peaks at 1.73 (a), 1.92 (b), 3.67 (c) and 4.06 (d) ppm were attributed to the protons of CH_2_ of the chlorobutane, respectively. The signals ranging from 7.71–7.95 (l) ppm belonged to protons of phenyl rings of TPP, which could be observed in TPP-Res. Despite a little shift of the Res related peaks due to the introduction of TPP, the characteristic peaks of TPP and Res were simultaneously observed in TPP-Res, which confirmed the formation of TPP-Res. The ^1^^3^C-NMR spectrum confirmed the aromatic peaks ranging from 120 to 140 ppm related to TPP and Res carbons of aromatic rings.

### 3.2. Preparation and Characterization of Liposomes

For the co-delivery system, it was essential to simultaneously encapsulate different agents in one carrier with an optimized drug ratio. In the current study, we prepared the hydrophilic drug doxorubicin hydrochloride (Dox·HCl) into the aqueous core using ammonium sulfate gradient method and the hydrophobic drug TPP-Res into the lipid layers using thin-film hydration method, respectively. We appropriately increased the pH value of ammonium sulfate and reduced the temperature of hydration to ensure the concentration of ammonium ions and the stability of liposomes [35].

**Figure 2:**
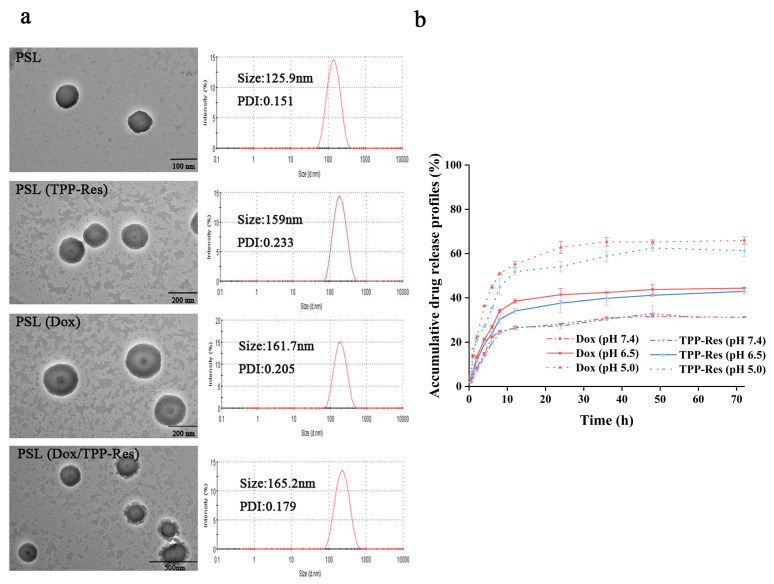
(**a**) Transmission electron microscope image and particle size distribution of different liposomes. (**b**) In vitro drug release of Dox and TPP-Res from PSL (Dox/TPP-Res) in PBS (pH 7.4, 6.5, 5.0). All data are expressed as mean values ± SD of 3 independent experiments.

The morphology of different formulations was observed using TEM. From the TEM images (Figure 2a), the liposomes showed a nearly spherical morphology with narrow size distribution. The particle size of blank pH-sensitive liposome (PSL) was ~125 nm with narrow PDI (~0.15). A similar size range with slight advance in particle size could be observed in drug-loaded liposomes. The particle size of PSL (Dox/TPP-Res) was ~165 nm, which was conducive to accumulate at the tumor site by EPR effect. As shown in Table 1, the zeta potential of the liposomes was mildly elevated, indicating that the cationic TPP was embedded in the bilayers of liposomes rather than being exposed on the surface of the liposomes. The encapsulation efficiency (EE %) of Dox and TPP-Res was approximately 90% and 70%, respectively. The results of all formulations were in close proximity, confirming that loading the two drugs in a single carrier did not affect their EE%.

To confirm the pH sensitivity of PSL (Dox/TPP-Res), we compared the release profile of the encapsulated Dox and TPP-Res from liposome at physiological pH 7.4, acidic pH 6.5 and pH 5.0 at 37 °C. As shown in Figure 2b, Dox and TPP-Res presented a steady sustained release from PSL (Dox/TPP-Res) at pH 7.4 with nearly 35% of drugs released, suggesting good stability of the liposome under physiological conditions. However, an appreciable increase in both payloads could be observed at pH 5.0 with the accumulative release of almost 65%. According to a previous study [36], an acidic pH environment could accelerate drug release, which was obviously attributed to the protonization of lipid membrane materials. At neutral pH, the structures of CHEMS and DOPE are complementary and form a stable bilayer structure. When the pH was lower at 5.0, the higher extent of protonated CHEMS probably tended to undergo a structural change from a bilayer structure to that of an inverted hexagonal (HII) structure, resulting in greater liposome structure damage and even faster drug release. In addition, the similar release profile of Dox and TPP-Res indicated that the liposome could be used as a carrier for simultaneous delivery of different hydrophobicity drugs.

### 3.3. Identification of Cancer Stem-Like Cells

**Figure 3:**
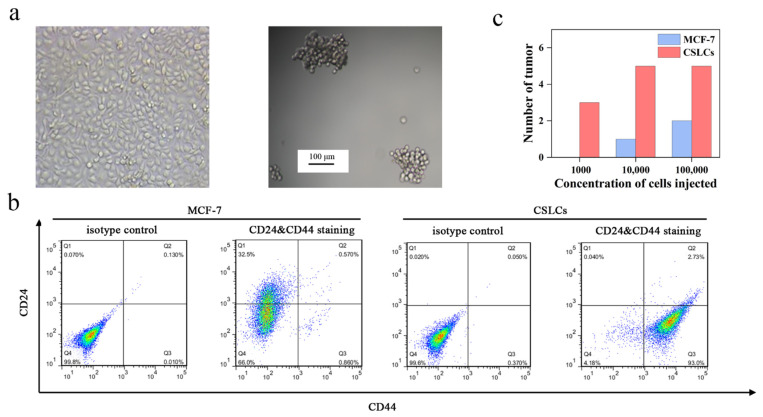
(**a**) Image of MCF-7 and CSLCs under the light microscope. (**b**) Identification of phenotype for MCF-7 and CSLCs stained with IgG-FITC and IgG-PE antibodies as isotype control and stained with anti-CD44-FITC and anti-CD24-PE antibodies. (**c**) The tumorigenic ability of MCF-7 and CSLCs transplanted into female nude mice.

We enriched CSLCs by culturing MCF-7 cells in serum-free medium and ultralow attachment plates similar to a previously reported method [37]. MCF-7 cells grew adherently in serum-containing medium (Figure 3a). Conversely, mammospheres were suspended in the medium and had a diameter of ~100 μm. MCF-7 cells with CD44^+^/CD24^−^ phenotypes have been demonstrated to have cancer stem-like cell tumor-initiating and invasive features [38,39]. Flow cytometry analysis of the mammospheres showed that CD44 was highly over-expressed in nearly 93% of the cells (Figure 3b). The expression of CD24 on the mammospheres was extremely low, but this receptor was highly expressed in MCF-7 cells (~32.5%). In addition, to verify the tumorigenic ability of mammospheres [40], we injected various amounts of MCF-7 cells and mammospheres into female nude mice. The results are shown in Figure 3c. Only 1000 mammospheres were required to give rise to new tumor masses while the same number of MCF-7 cells could not. Therefore, 1 × 10^4^ cells/mL mammospheres was used to establish the xenograft tumor model. These results indicated that the enriched non-adherent mammospheres had the characteristics of CSLCs.

### 3.4. Ratio-Dependent Synergy of a Dox and TPP-Res Combination In Vitro

**Figure 4:**
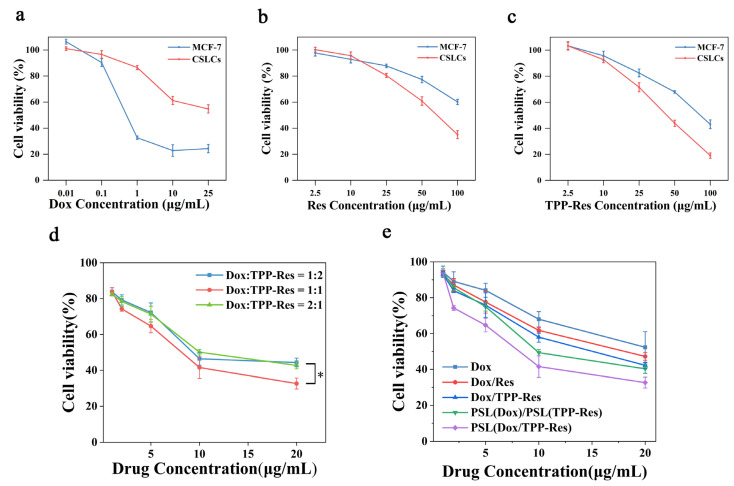
In vitro cytotoxicity of DOX (**a**), Res (**b**) and TPP-Res (**c**) on the MCF-7 and CSLCs; (**d**) cytotoxicity of three weight ratios (1:2, 1:1 and 2:1) of Dox and TPP-Res in the liposomes on the CSLCs. * *p* < 0.05 compared with the ratio of 1:1 group. (**e**) Cytotoxicity of different formulations at a Dox/TPP-Res ratio of 1:1 on the CSLCs. Cell viability (%) was expressed as a percentage compared to the untreated control cells. All data are expressed as mean values ± SD of 5 independent experiments.

Prior to of the cytotoxicity of Dox combined with TPP-Res against CSLCs, we first assayed the resistance characteristic of CSLCs. The IC_50_ of Dox was calculated to be ~1.05 ± 0.01 μg/mL on MCF-7 but was 28.33 ± 0.66 μg/mL on CSLCs (Figure 4a), proving that CSLCs enriched by the serum-free culture method equipped higher resistance to Dox than MCF-7. To substantiate the apoptosis-inducing effect of Res, the cytotoxicity of TPP-Res and Res against CSLCs and MCF-7 was determined (Figure 4b,c). A marked reduction in MCF-7 and CSLCs was observed in a concentration-dependent manner. In addition, TPP-Res (IC_50_ = 36.14 ± 0.86 μg/mL) evoked more potent cytotoxicity than Res (IC_50_ = 65.88 ± 1.57 μg/mL) in CSLCs. This was probably due to the higher positive charge of cationic TPP, which increased cell uptake of Res and induced mitochondrial apoptosis.

To evaluate the cytotoxicity of Dox combined with TPP-Res against CSLCs, the cytotoxicities of PSL (Dox/TPP-Res) encapsulating three different drug weight ratios (1:2, 1:1 and 2:1) were examined against CSLCs, while keeping the total drug mass encapsulated constant. As shown in Figure 4d, the IC_50_ of Dox combined with TPP-Res (1:1) decreased to 14.30 ± 0.11 μg/mL, suggesting that TPP-Res potentiates the cytotoxic effect of Dox on the CSLCs. Importantly, the cytotoxicity of Dox/TPP-Res at the ratio of 1:1 significantly increased compared to that of 1:2 and 2:1 (*p* < 0.05). Therefore, we selected a weight ratio of 1:1 for the following study.

The cell viability and IC_50_ values of different formulations at a Dox/TPP-Res ratio of 1:1 against CSLCs are shown in Figure 4e and Table 2. PSL (Dox/TPP-Res) exhibited much lower IC_50_ and higher RF compared to other groups, highlighting the significance of the synergistic effect of Dox and TPP-Res.

Collectively, the concentration of Dox and TPP-Res was set to a weight ratio of 1:1 (10 μg/mL) for the following study due to the moderate cytotoxicity. At this concentration, Dox showed significant drug resistance to CSLCs, and the cell viability of TPP-Res in both MCF-7 and CSLCs were above 80%, which were convenient to study the reversal effect.

### 3.5. Cellular Uptake of Dox

**Figure 5:**
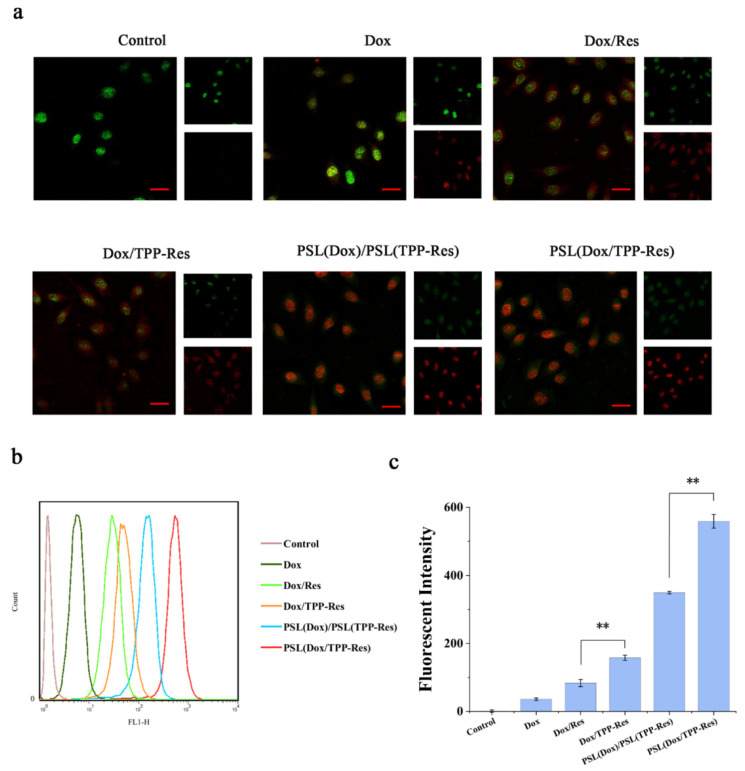
(**a**) Fluorescence microscopy images of CSLCs incubated with different formulations (Red: DOX; Green: CSLCs labeled with anti-CD44-FITC). (**b**,**c**) Flow cytometry measurements of the intracellular uptake of Dox in CSLCs treated with different formulations. Data were expressed as mean ± SD (n = 3), ** *p* < 0.01 compared with the control group. Scale bars represent 50 μm.

To assess the property of the liposome delivery to CSLCs, fluorescence microscopy and a flow cytometer were separately used to measure the distribution of Dox. To visualize CSLCs, cells were labeled with the anti-CD44-FITC (green fluorescence). As depicted in Figure 5a, the presence of TPP-Res and Res markedly increased the intracellular influx of Dox, suggesting that Res played an important role in reversing the drug resistance of CSLCs. The liposome groups (PSL (Dox)/PSL (TPP-Res) and PSL (Dox/TPP-Res)) showed a stronger red fluorescence than free drugs, indicating that the protection of liposomes could prevent the degradation of endosomes.

The fluorescence of Dox in CSLCs analyzed by flow cytometry corroborated the similar uptake tendency (Figure 5b,c). Furthermore, the PSL (Dox/TPP-Res) group was prominently more effective than the PSL (Dox)/PSL (TPP-Res) group, indicating that the co-delivery liposomes could better enable the synchronous release of both drugs for generating their synergistic effect than physical mixing liposomes.

### 3.6. Intracellular Trafficking

**Figure 6:**
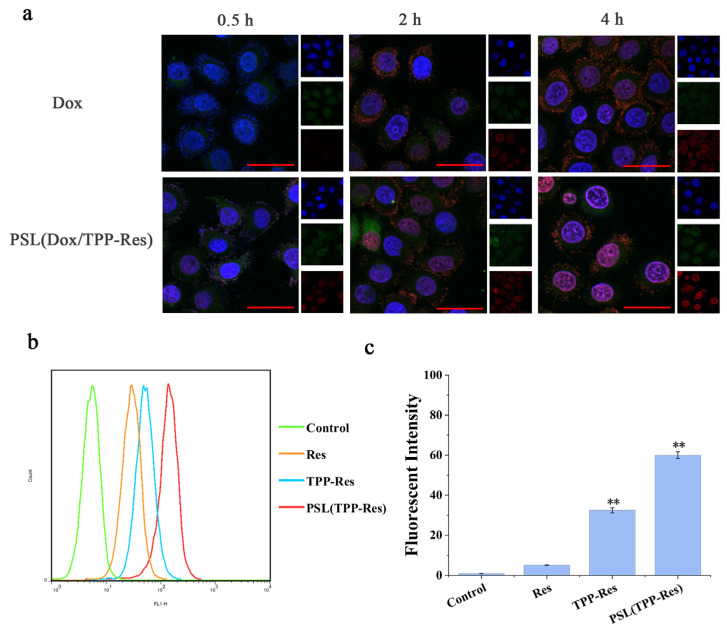
(**a**) Confocal laser scanning microscopy images of CSLCs incubated with PSL (Dox/TPP-Res) and free Dox solution for 0.5 h, 2 h and 4 h (Red: DOX; Green: lysotracker-Green; Blue: Honchest 33258). (**b**,**c**) Flow cytometry measurement of TPP-Res in mitochondrial fraction in CSLCs treated with different formulations. Data were expressed as mean ± SD (n = 3), ** *p* < 0.01 compared with the control group. Scale bars represent 50 μm.

Endosomal degradation of the therapeutic agent was one of the major issues associated with the nanoparticle drug delivery system. The nanoparticles were internalized in the cells via different endocytosis pathways. Then, they were delivered from early endosomes to the late endosome and to the lysosome. During the process, nanoparticles encountered a harsh chemical environment resulting in the degradation of nanoparticles and their content. To evaluate endosomal escape ability of the liposomes, confocal laser scanning microscopy was used to observe the subcellular distribution of Dox. As illustrated in Figure 6a, we labeled the nucleus with blue fluorescence (Hoechst 33258) and the endosome with green fluorescence (LysoTracker DND-26). PSL (Dox/TPP-Res) showed weak red fluorescence around the nucleus after 30 min, indicating that Dox accumulated in endosomes after endocytosis. The red fluorescence of Dox increased gradually during incubation, which suggested the continuous accumulation of the liposomes in the endosomes. After incubation for 4 h, PSL (Dox/TPP-Res) revealed a strong purple fluorescence (overlap of Dox with Hoechst 33258), indicating that Dox had escaped from endosomes into nuclei. In contrast, free Dox showed weak red fluorescence in the endosomes only, indicating that Dox was degraded in the endosomes due to the drug-resistance effect in CSLCs. These results suggested that the pH-sensitive liposome delivery system and the synergism of TPP-Res improved the uptake of Dox in the nucleus and reversed the resistance in CSLCs.

Since the liposome escaped from the endosome, TPP-Res was exposed in the cytoplasm. To assess the mitochondrial-targeting efficiency of TPP-Res, we detected the uptake of Res in the mitochondrial fraction. Mitochondria were isolated by a mitochondria extraction kit and the fluorescence intensity of CSLCs was measured by flow cytometry. As shown in Figure 6b,c, there was a remarkable increase in PSL (TPP-Res) compared with Res and TPP-Res, which suggested that the liposomes increased drug accumulation into cytoplasm and TPP-Res effectively targeted mitochondria. The above results indicated that the hierarchical targeted drug delivery system achieved accurate delivery by effective endosomal escape, mitochondrial targeting and synergistic effects of drugs [41].

### 3.7. Cell Apoptosis Assay

Apoptosis is a process of programmed cell death involved in cellular stress responses. It has been shown that mitochondria play crucial roles in triggering apoptosis through reducing mitochondrial membrane potential, decreasing ATP production and activating caspase cascade reaction. To determine whether PSL (Dox/TPP-Res) would effectively target mitochondria and induce cell apoptosis, we examined the following apoptosis markers against CSLCs.

**Figure 7:**
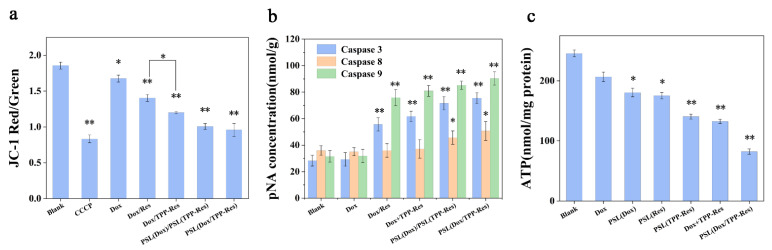
(**a**) The effect on the mitochondrial membrane potential in the CSLCs incubated with different formulations. (**b**) Activity of caspase-3, caspase-8 and caspase-9 in the CSLCs incubated with different formulations. (**c**) Intracellular ATP in the CSLCs incubated with different formulations. Data were expressed as mean ± SD (n = 3), * *p* < 0.05, ** *p* < 0.01 compared with the blank group.

The decrease of mitochondrial membrane potential could be used as an early signal of apoptosis. CCCP (carbonyl cyanide 3-chlorophenylhydrazone) was a potent inhibitor of coupled electron transport, which leads to the loss of membrane potential on both sides of the mitochondrial inner membrane, as a positive control. As depicted in Figure 7a, the mitochondrial membrane potential of CSLCs dramatically decreased after incubation with Dox/TPP-Res compared with Dox/Res, indicating that TPP-Res was rapidly enriched in mitochondria under the cationic TPP (*p* < 0.05). PSL (Dox/TPP-Res) evidently triggered the dissipation of ΔΨm in CSLCs, which may be due to the rapid binding of TPP-Res to mitochondria after entering into cytoplasm from endosome.

The caspase family, as key executors of apoptosis, was also detected. As shown in Figure 7b, caspase-9 and caspase-3 activities were considerably increased after treatment with PSL (Dox/TPP-Res), reflecting that the apoptosis induced by PSL (Dox/TPP-Res) was principally related to the mitochondria-mediated apoptosis pathway attributed to TPP-Res (*p* < 0.01). After the mitochondrial membrane permeabilization, cytochrome C was released into the cytosol. Caspase-9 was activated by the complex of Apaf-1, procasepase-9 and cytochrome C, resulting in initiating caspase-3 [42]. In addition, PSL (Dox/TPP-Res) changed the activity of caspase-8 which is related to the fas apoptosis pathway (*p* < 0.05). This result demonstrated that PSL (Dox/TPP-Res) may induce further proteolytic cascades and ultimately cause CSLCs death.

To investigate the effect on mitochondrial function, the ATP levels of CSLCs were measured (Figure 7c). The ATP content of CSLCs in PSL (Dox/TPP-Res) was 82.2 ± 9.64 nmol/mg protein, which was lower than other groups. This result indicated that PSL (Dox/TPP-Res) destroyed the normal activity of the ATP metabolism in mitochondria, which then could inhibit drug efflux and induce apoptosis. To summarize, PSL (Dox/TPP-Res) had the ability to target mitochondria and induce apoptosis via the mitochondria pathway.

### 3.8. Suppress Wnt/β-Catenin Signaling Pathway

**Figure 8:**
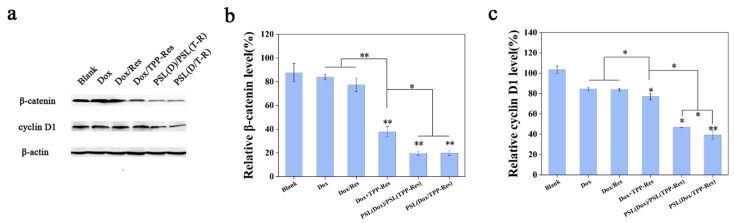
(**a**) Western blotting analysis of β-catenin and cyclin D1 in CSLCs treated with different formulations. (**b**) β-catenin and (**c**) cyclin D1 relative to β-actin were determined by Image J. Data were expressed as mean ± SD (n = 3), * *p* < 0.05, ** *p* < 0.01 compared with the blank group.

Aberrant activation of Wnt/β-catenin signaling pathway was primarily responsible for tumor initiation and tumor sphere formation [43]. To inspect this effect, the expression levels of β-catenin and cyclin D1 were evaluated using Western blotting. As shown in Figure 8a–c, PSL (Dox/TPP-Res) significantly decreased the expression of β-catenin and cyclin D1 compared to the control group. The effect of TPP-Res on the Wnt/β-catenin signaling pathway further validated its inhibitory effect on CSLCs [44].

### 3.9. Biodistribution in CSLCs Xenograft Nude Mice

**Figure 9:**
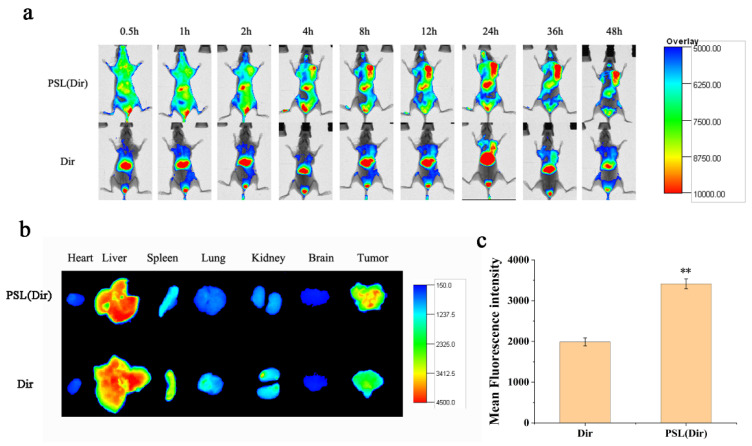
(**a**) In vivo fluorescent images at different times after intravenous injection of PSL (Dir) and Dir. (**b**) Tissue fluorescent images at 48 h post-injection in heart, liver, spleen, lung, kidney, brain and tumor. (**c**) The fluorescence intensity of tumor regions was analyzed using NIH Image J software. Data were expressed as mean ± SD (n = 3), ** *p* < 0.01 compared with the Dir group.

To evaluate the distribution status of the liposomes, in vivo imaging observation was performed. Dir was used as a probe and was encapsulated into the liposome to indicate the distribution. As shown in Figure 9a, the fluorescence of PSL (Dir) was first concentrated in the liver and gradually increased in the tumor site. After 48 h, the tumor masses still showed fluorescence. This result suggested that the liposomes persisted in the blood circulation and exhibited the enhanced permeability retention (EPR) effect. After the mice were sacrificed, the tumors and major organs were isolated. Figure 9b,c showed that the liposomes displayed higher Dir accumulation in tumors and lower accumulation in liver and spleen than free Dir, while no other major organs seemed to be affected. Therefore, the pH-sensitive liposome was believed to have potential for tumor targeting and to be efficient for payloads delivery.

### 3.10. In Vivo Antitumor Effect

**Figure 10:**
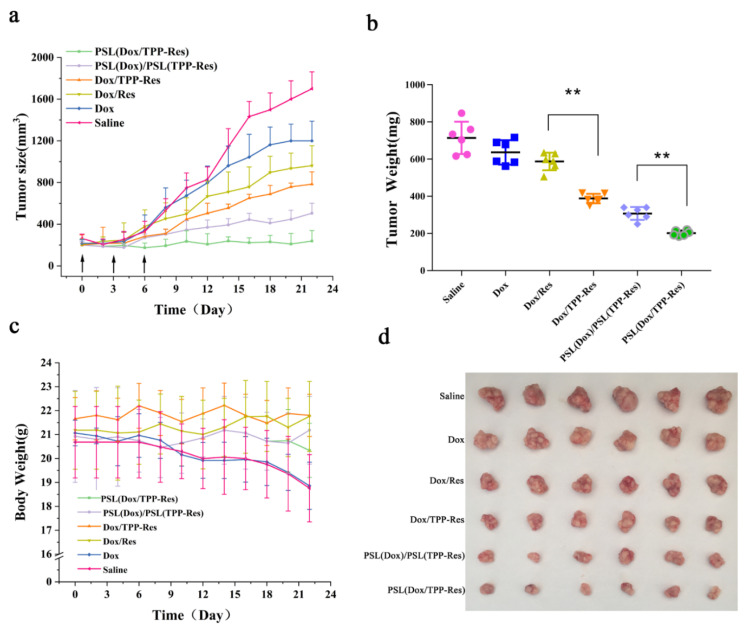
(**a**) Tumor size, (**b**) tumor weight and (**c**) body weight of the CSLCs xenograft tumor-bearing nude mice (n = 6) after intravenous injection with different formulations. (**d**) The images of excised tumor tissues at the time of sacrifice. Data were expressed as mean ± SD (n = 6), ** *p* < 0.01 compared with the control group.

The in vivo antitumor effect of liposomes was investigated using a breast malignant tumor model established through inoculating CSLCs into female nude mice. As shown in Figure 10a,b, the anticancer effect of Dox group was modest due to the resistance of CSLCs. Compared with the Dox/Res group, the addition of TPP observably improved the inhibition efficiency (*p* < 0.01), which could be attributed to their mitochondria targeting. PSL (Dox/TPP-Res) had a greater anticancer effect than PSL (Dox)/PSL (TPP-Res) (*p* < 0.01), revealing the prominent role of synergistic ratiometric delivery of Dox and TPP-Res through liposomes. Meanwhile, all groups displayed no obvious loss in body weight, except the free Dox and control, indicating the good compatibility of the liposomes (Figure 10c).

## 4. Conclusions

In this study, novel mitochondria-targeted derivate TPP-Res was synthesized and incorporated with Dox into the pH-sensitive liposomes to conquer the cancer stem cells-associated therapeutic obstacle. PSL (Dox/TPP-Res) was characterized to have a small particle size, high encapsulation efficiency and excellent pH-sensitive characteristics. The cellular uptake and intracellular trafficking indicated that PSL (Dox/TPP-Res) facilitated effective endo-lysosomal escape, mitochondrial targeting and synergistic effects of drugs. The mechanisms for the enhanced anticancer efficacy in CSLC were due to decreasing of mitochondrial membrane potential, activation of caspase, reduction of ATP level and suppression of the Wnt/β-catenin pathway. Furthermore, the liposomes had a satisfactory accumulation in tumor in vivo, which provides possibilities for practical application. The present study provides a promising strategy for a nanotherapeutic approach to overcome the CSC-associated chemotherapeutic resistance.

## Figures and Tables

**Table 1:** Characterization of different liposomes.

Formulation	Particle Size (nm)	Zeta Potential (mV)	EE%	
			Dox	TPP-Res
PSL	125.9 ± 1.6	−26.5	-	-
PSL (TPP-Res)	154.6 ± 3.5	−20.4	-	69.2 ± 6.28
PSL (Dox)	158.3 ± 4.1	−21.3	96.8 ± 0.18	-
PSL (TPP-Res/Dox)	165.2 ± 5.7	−19.2	94.5 ± 0.32	68.5 ± 1.74

**Table 2:** The IC_50_ and resistance factor (RF) on the CSLCs.

Formulation	IC_50_ (μg/mL)	RF
Dox	28.33 ± 0.66	-
Res	65.88 ± 1.57	-
TPP-Res	36.14 ± 0.86	-
Dox/Res	17.56 ± 0.14	1.61
Dox/TPP-Res	14.30 ± 0.11	1.98
PSL (Dox)/PSL (TPP-Res)	12.16 ± 0.05	2.33
PSL (Dox/TPP-Res)	8.33 ± 0.07	3.40

## Data Availability

The data presented in this study are available on request from the corresponding author.

## Supplementary Materials

The following are available online at https://www.mdpi.com/article/10.3390/pharmaceutics13081205/s1, Figure S1: Synthetic route of TPP-Res, Figure S2: (a) ^1^H-NMR spectrum of TPP-Res; (b) ^13^C-NMR spectrum of TPP-Res, Figure S3: ^1^H-NMR spectrum of Res (a), 4′-(4-O-chlorobutyl) resveratrol (b) and 4′-(4-O-iodobutyl) resveratrol (c).
