# Supplementary material for: Construction of Hierarchical-Targeting pH-Sensitive Liposomes to Reverse Chemotherapeutic Resistance of Cancer Stem-like Cells
Source: Pharmaceutics. 2021 Aug 5;13(8):1205. doi: 10.3390/pharmaceutics13081205 (PMC8399523; doi:10.3390/pharmaceutics13081205)
Supplement: Supplementary file 1 [file pharmaceutics-13-01205-s001.zip › pharmaceutics-1250248-supplementary.pdf]

pharmaceutics

MDPI

Supplementary Materials: Construction of Hierarchical-Targeting pH-Sensitive Liposomes to Reverse Chemotherapeutic Resistance of Cancer Stem-Like Cells

Shuang Ba, Mingxi Qiao, Li Jia, Jiulong Zhang, Xiuli Zhao, Haiyang Hu and Dawei Chen

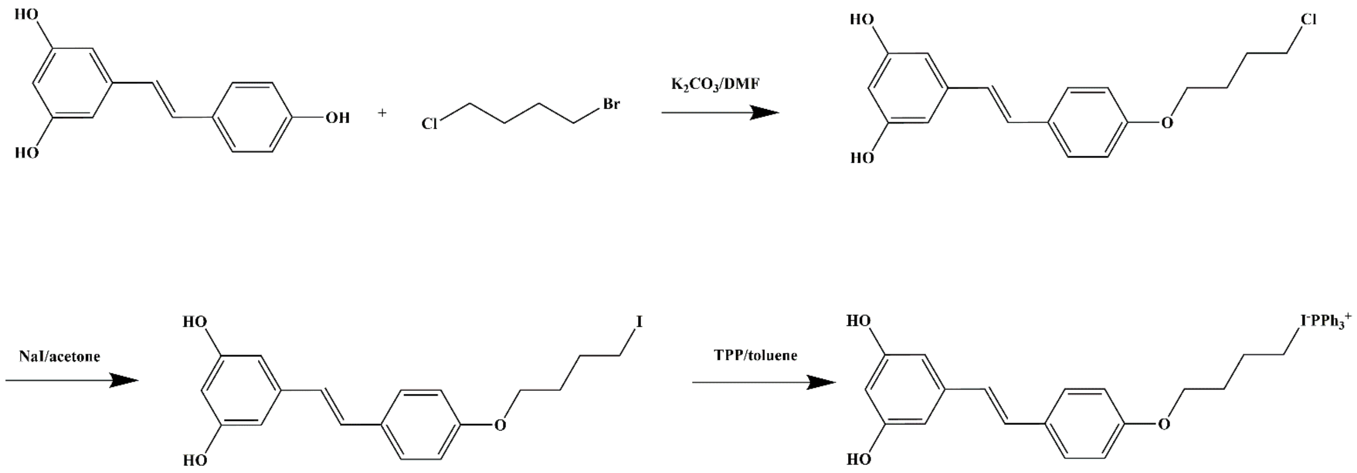

Figure S1. Synthetic route of TPP-Res.

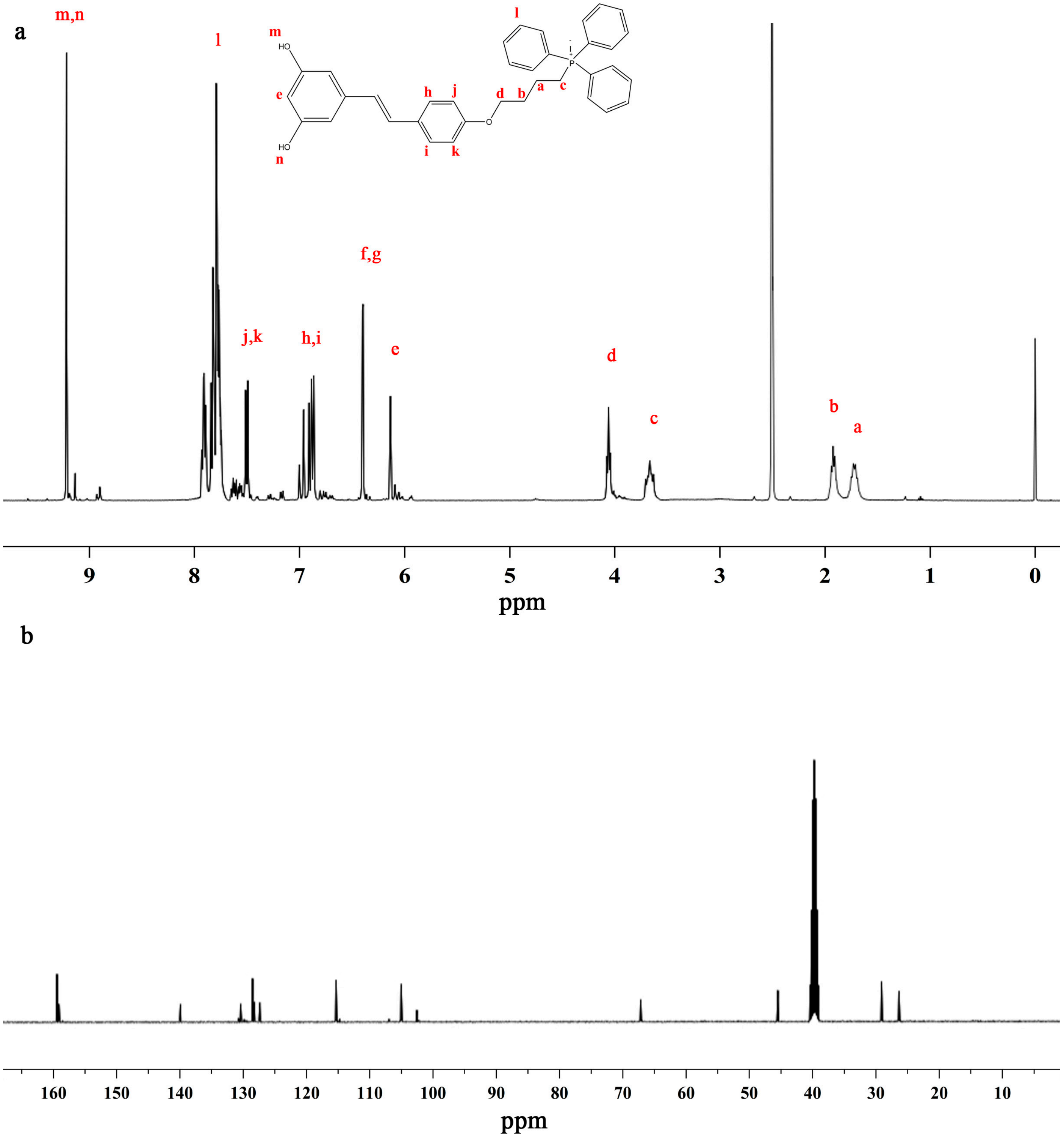

Figure S2. (**a**) ^{1}H-NMR spectrum of TPP-Res; (**b**) ^{13}C-NMR spectrum of TPP-Res.

The ^{1}H-NMR spectrum of TPP-Res exhibited the following absorptions(ppm):1.73 (quintet, 2H, CH_{2TPP}), 1.92 (quintet, 2H, CH_{2TPP}),3.67 (t, 2H, CH_{2TPP}), 4.06 (t, 2H, CH_{2TPP}), 6.13 (t, 1H, H_{Res}-4, J = 1.9 Hz), 6.40 (d, 2H, H_{Res}-2,H_{Res}-6, J=1.75 Hz), 6.83–7.04 (m, 4H, =CH, H_{Res}-2', H_{Res}-6'), 7.50 (d, 2H, H_{Res}-3', H_{Res}-5', J=8.75 Hz), 7.71–7.95 (m, 15H, aromatic-H_{TPP}), 9.22 (s, 2H, 3 -OH_{Res}, 5 -OH_{Res}).

The ^{13}C-NMR spectrum of TPP-Res exhibited the following absorbtions(ppm):26.14, 29.15, 40.19, 45.19, 65.93, 104.38, 114.66, 126.67, 127.41, 127.73, 129.72, 130.25, 139.03, 158.00, 158.49.

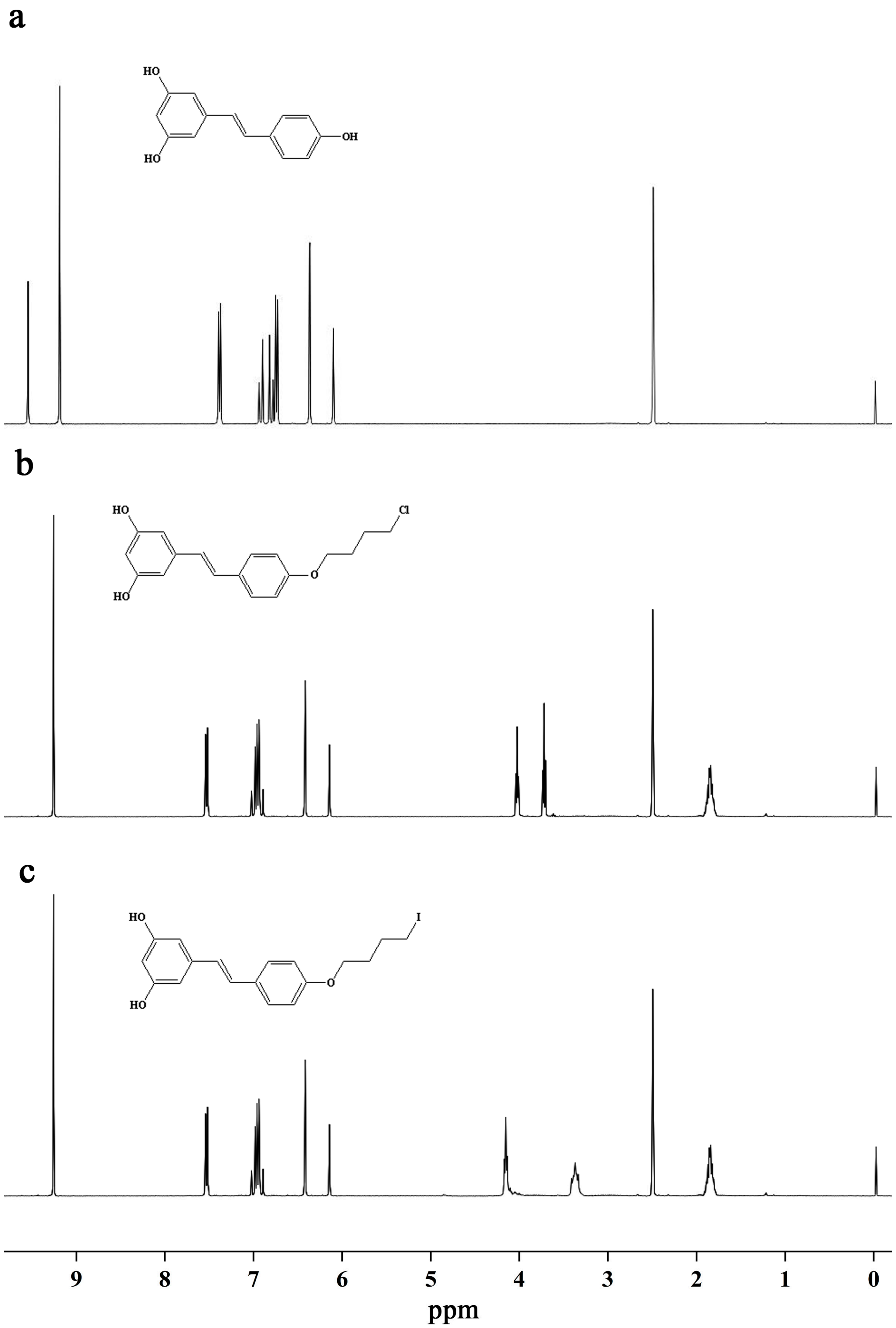

Figure S3. ^{1}H-NMR spectrum of Res(**a**), 4'-(4-O-chlorobutyl) resveratrol(**b**) and 4'-(4-O-iodobutyl) resveratrol(**c**).

The ^{1}H-NMR spectrum of Res exhibited the following absorptions(ppm): 6.12 (t, 1H, H_{Res}-4, *J* = 2.0 Hz), 6.40 (d, 2H, H_{Res}-2,H_{Res}-6, J = 2.0 Hz),6.75(m, 4H, =CH, H_{Res}-2', H_{Res}-6'), 7.38 (d, 2H, H_{Res}-3', H_{Res}-5', J = 8.75 Hz), 9.07 (s, 2H, 3 -OH_{Res}, 5 -OH_{Res}), 9.68(s, 1H, 4′-OH_{Res}).

The ^{1}H-NMR spectrum of 4'-(4-O-chlorobutyl) resveratrol exhibited the following absorptions(ppm): 1.76–1.98 (m, 4H, CH_{2TPP}), 3.72(t, 2H, CH_{2TPP}), 4.02 (t, 2H, CH_{2TPP}), 6.12 (t, 1H, H_{Res}-4, *J* = 2.0 Hz), 6.40 (d, 2H, H_{Res}-2,H_{Res}-6, *J* = 2.0 Hz,), 6.82–7.04 (m, 4H, =CH, H_{Res}-2', H_{Res}-6'), 7.50 (d, 2H, H_{Res}-3', H_{Res}-5', J = 8.75 Hz), 9.21 (s, 2H, 3 -OH_{Res}, 5 -OH_{Res});

The ^{1}H-NMR spectrum of 4'-(4-O-iodobutyl) resveratrol exhibited the following absorptions(ppm): 1.71–2.00 (m, 4H, CH_{2TPP}), 3.35 (t, 2H, CH_{2TPP}), 4.01 (t, 2H, CH_{2TPP}), 6.12 (t, 1H, H_{Res}-4, *J* = 2.0 Hz), 6.40 (d, 2H, H_{Res}-2,H_{Res}-6, J = 2.0 Hz), 6.82–7.04 (m, 4H, =CH, H_{Res}-2', H_{Res}-6'), 7.50 (d, 2H, H_{Res}-3', H_{Res}-5', J = 8.75 Hz), 9.21 (s, 2H, 3 -OH_{Res}, 5 -OH_{Res}).
